# Sclerosing Microcystic Adenocarcinoma of the Base of Tongue with Signet Ring-Like Cell Component: A Rare Case

**DOI:** 10.30699/IJP.2023.1989943.3068

**Published:** 2023-12-29

**Authors:** Parisa Mokhles, Alireza Sadeghipour, Maryam Kadivar, Nasrin Shayanfar, Zahra Keshtpour Amlashi, Pegah Alizade Pahlavan, Ashkan Shafiei

**Affiliations:** 1 *School of Medicine, Iran University of Medical Sciences, Tehran, Iran *; 2 *School of Medicine, Hamedan University of Medical Sciences, Hamedan, Iran*; 3 *Skull Base Research Center, Five Sense Health Institute School of Medicine, Iran University of Medical Sciences, Tehran, Iran*

**Keywords:** Adenocarcinoma, Head and neck neoplasm, Salivary gland

## Abstract

Sclerosing microcystic adenocarcinoma (SMA) is an uncommon neoplasm of the oral cavity, with only 14 reported cases documented in the literature.

We present a case of SMA in a 65-year-old woman with a history of high-grade lymphoma who developed clear cell changes in the deep muscular layer of the tongue.

Currently, the diagnosis of SMA relies on careful morphological evaluation and the exclusion of other potential differential diagnoses.

## Introduction

Sclerosing microcystic adenocarcinoma (SMA) is a rare oral cavity neoplasia that shares histological similarities with microcystic adnexal carcinoma (MAC) of the skin. Despite its clinical significance, SMA is not recognized in the latest WHO classification. Only fourteen cases of SMA have been reported in the literature, often observed in the context of immunosuppression and prior malignancies. Initially referred to as a MAC-like tumor arising at the mucosal surfaces of the head and neck, SMA is believed to originate from the minor salivary glands in these regions, given the absence of adnexal glands in the mucosal layers (1, 2).

Microscopically, SMA is characterized by infiltrating nests and ducts of cytologically bland-looking cells intermixed with prominent stromal components. The stroma exhibits various features, from densely collagenized to basophilic desmoplasia, frequently surrounding the ducts. There is no known molecular alteration of SMA, and the immunohistochemistry profile is nonspecific. Therefore, the diagnosis relies on meticulous morphological evaluation, posing challenges in cases where diagnostic features are not readily evident, particularly in small incisional biopsies (3). 

This article presents a case of SMA, representing the third reported instance of this extremely rare neoplasm located at the base of the tongue, with unique signetring-like apparent cell changes observed at the muscular layer.

## Case Presentation

The patient was a 65-year-old non-smoking woman who presented with a tongue mass that had been present for the last two months. She experienced dysphagia for solids one year before the presentation, with no reported prior injury to the area. Thirty years ago, she was admitted due to a right-sided cervical mass and received 76 Gy/35 fx external radiation followed by a CHOP chemotherapy regimen for a diagnosis of poorly differentiated nasopharyngeal lymphoma. During the intraoral physical examination, an ulcerated rough mass was observed in the left posterior base of the tongue, crossing the midline and extending to the right side without any tenderness, bleeding, or secretion.

**Fig. 1:**
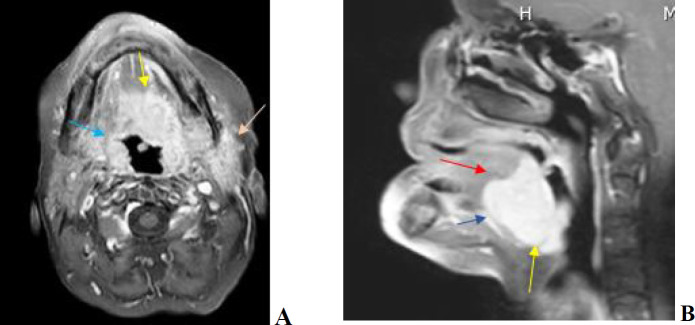
(a) MRI of the head and neck shows one large infiltrative enhancing mass arising from the left posterior aspect of the tongue (b), which crosses the midline and extends to the oropharyncous, left parapharyngeal space, left masticator, and parotid space

A head and neck MRI revealed a large infiltrative enhancing mass originating from the left posterior aspect of the tongue (Figure 1a, b). The mass extended to the oropharynx, left parapharyngeal space, left masticator, and parotid space. It crossed the midline and extended to the right portion of the tongue, with additional extensions noted to the left palatine tonsil, left vallecula, and sublingual muscles. The MRI also showed neurovascular bundle encasement, but no cervical lymphadenopathy was observed.

**Fig. 2:**
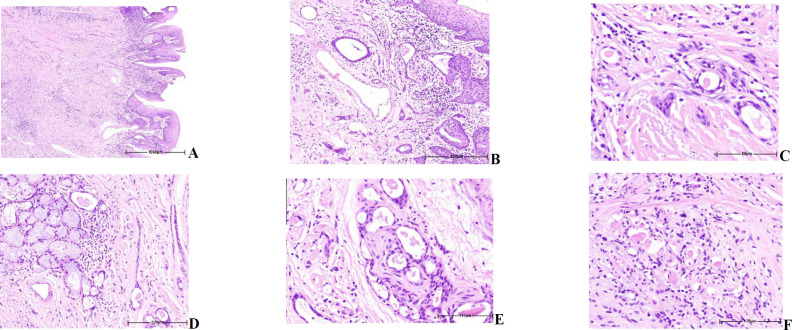
Histologic sections showing a pseudoepitheliomatous hyperplastic epithelium without atypia (a. H&E. x40), lamina propria is diffusely infiltrated by a pauci-cellular, ill-defined neoplastic infiltration with isolated tubular and cord structures (b. H&E. x100). The neoplasm is composed of small cuboidal to spindle bland-looking mitotically inactive cells forming microcystic lumina, frequently filled by eosinophilic secretions, embedded in a partially sclerotic stroma (c. H&E. x600), in close association with minor local salivary glands (d. H&E. x100) showing neural invasion (e. H&E. x400). Deeper portions of the neoplasm mainly consist of signet ring-like cells with bland-looking nuclei, infiltrating interlacing striated muscle bundles (f. H&E. x400)

**Fig. 3:**
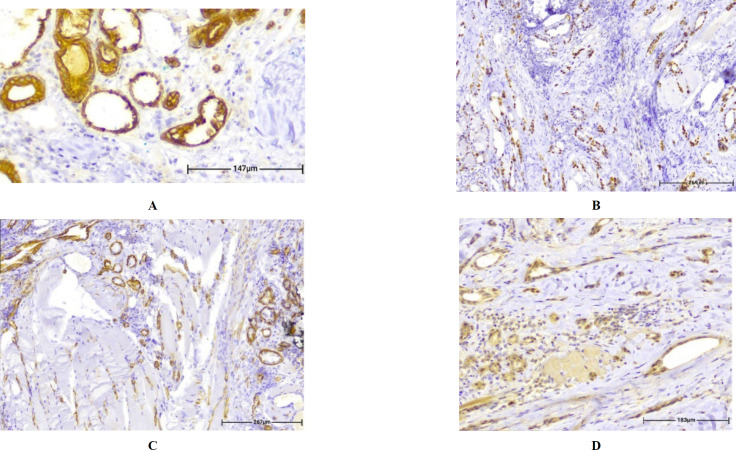
By immunostaining, the tumoral cells were positive for CK7 (a. IHC. X400), smooth muscle actin (b. IHC. X100), P63 (c. IHC. X100), and P40 (d. IHC. X200) with a pattern similar to the epithelium-myoepithelium in both superficial and deep portions of the neoplasm

An incisional biopsy was performed. Histologic sections demonstrated an ill-defined submucosal neoplastic infiltration composed of relatively bland-looking small cuboidal to spindle-shaped and mitotically inactive tumoral cells containing mildly pleomorphic nuclei arranged in discrete cords, isolated tubules and bilayer strands of epithelioid cells forming micro-cystic lumina frequently filled by eosinophilic secretions, embedded in a partially sclerotic stroma (Figure 2c). The epithelial lining showed pseudoepitheliomatous hyperplasia without atypia (Figure 2a, b). Deeper portions of the neoplasm predominantly consisted of signet ring-like cells with bland-looking nuclei arranged in cords, nests, and small ductal structures infiltrating the interlacing striated muscle bundles of the tongue parenchyma (Figure 2f). By immunostainings, the tumoral cells were positive for Ck7 in luminal cells and for smooth muscle actin (SMA), P63, and P40 in non-luminal cells, similar to the epithelium-myoepithelium pattern in many other salivary gland tumors at both superficial and deep portions of the neoplasm as well as signet ring-like cells (Figure 3, a, b, c, d). CD117 (C-kit) was weakly positive in less than 10% of the luminal cells analogous to the normal salivary gland epithelial cells and interpreted as a negative result. Ki-67 showed positive nuclear staining in less than 5% of tumoral cells. The neoplasm was closely associated with minor salivary glands (Figure 2e). All surgical margins were involved and perineural invasion was noted. (Figure 2d).

The histomorphologic and immunostaining results strongly supported the final diagnosis of sclerosing microcystic adenocarcinoma. However, the patient did not provide consent for the excisional surgery.

**Table 1:** Previously reported SMA cases with brief clinical and IHC features

	Author	Age	Location	Positive IHC	Negative IHC	Medical history
1	Schipper* et al.* (1), 1995	65M	Tongue	CAM5.2, CEA, EMA	Not mentioned	Not reported
2	Petersson* et al.* (2), 2009	70F	Left posterior tongue	CK7, LMWCK, BerEP4, HMWCK, CK5, CK18, P63	Not mentioned	Not reported
3	Wood* et al.* (8), 2018	68F	Tip of tongue	CK7, CAM5.2, P63, S100	CK20, CD117, ER, PR, TTF-1, CDX2, SMA, Calponin, Ki-67 <5%	Not reported
4		49F	Right lateral tongue	CK7, P63, S100, CK5/6	ER, PR, TTF-1, CDX-2, Calponin, CK20, SMA, Ki-67 <5%	MPGN, Cystadenoma of ovary
5	Mills* et al.* (7), 2016	41F	Tongue base	Not mentioned	CD117	ACC underwent RT
6		47F	Anterior tongue	Not mentioned	Not mentioned	Not reported
7		73M	Nasopharynx, clivus	BerEP4, S100	CD117	Not reported
8		54F	Floor of mouth	CK, SMA	Not mentioned	Not reported
9		48F	Floor of mouth	CK5/6	CK7, Ki67: 5%	AML
10	Zhang* et al.* (3), 2019	55F	Floor of mouth	AE1/3, CK5/6, CK7, EMA, P63, P40, S100	AR, β-catenin, CD68, IgG4, CD117, SOX10, CD34	MS, BRCA positive
11	Zhang* et al.* (14), 2020	51M	Right tongue tip	CK5/6, CK8/18, EMA, CK7, P63, S100, CD10, SMA	CK20, Calponin, MYB, bcl2, p53, CD117, CD43, Ki67<5%, HER2	Not reported
12	Jiang* et al.* (5), 2020	41F	Left tongue	CK7, P63, P40	SOX10, CD117, S100, Mammoglobin, GATA3	Psoriatic arthritis underwent IT
13	G.Z.Tan* et al.* (9), 2021	73M	Left parotid	EMA, CK7, SOX10, P63, S100	CD117, Ki67: 5%	NPC underwent RT, Concurrent tonsillar SCC
14	Lee YiY* et al.* (4), 2022	48F	Left tongue	Ck7, P63, P40	CD117, Ki67<5%	Right tongue SCC
15	Current case	65F	Left posterior tongue	Ck7, P63, P40, SMA	CD117, Ki67<5%	High-grade lymphoma underwent RT

BerEp4, Ep-CAM5.2: Epithelial specific antigen; CEA: Carcinoembryonic antigen, CK: Cytokeratin, EMA: Epithelial membrane antigen, ER: Estrogen receptor, HMWCK: High molecular weight cytokeratin, LMWCK: Low molecular weight cytokeratin, MYB: Avian MYB viral oncogene homolog, PR: Progesterone receptor, SMA: Smooth muscle actin, SOX10: SRY-related HMG-box 10, MPGN: Membranoproliferative glomerulonephritis, ACC: Adenoid cystic carcinoma, RT: Radiotherapy, AML: Acute myeloid leukemia, MS: Multiple sclerosis, IT: Immunosuppressive therapy, NPC: Nasopharyngeal carcinoma.

**Table 2:** Possible differential diagnoses of SMA and their diagnostic key features

Category	Differential diagnosis	Microscopic descriptions		Main differential markers	Main molecular test
Non-neoplastic	Chronic sclerosing sialadenitis	Lacks sclerotic stroma or invasive growth pattern.		As normal histology	-
Neoplastic	Adenoid cystic carcinoma	-Angulated hyperchromatic nuclei -Containing basophilic secretions. (15)		CD 117 + P63+ P40+	MYB-NFIB fusion (16)
	Polymorphous adenocarcinoma (PAC)	-Well circumscribed at low power view. -More cellular. -Shows architectural variability. (17)		S100+ P63+ P40-	PRKD1 (18)
	Neoplasms with clear cell/signet ring features	Signet ring cell adenocarcinoma	-Mainly composed of cells with signet ring morphology arranged in islands, cords, etc. -No myoepithelial differentiation. (10)	P63+ Pan Ck+ SMA -	Recurrent AKT1-E17K Mutations (19)
		Hyalinizing clear cell carcinoma	-Nests of clear epithelial cells. -No myoepithelial differentiation. -Cellular fibrohyalinized septation. (20)	Ck5/6+ P63+ SMA – S100-	EWSR-ATF1 fusion (20)
		Low-grade mucoepidermoid carcinoma	-Often cystic -Admixture of epidermoid, squamoid, intermediate, and mucinous cells. (21)	P63+ (in intermediate cells) P40+ Ck7,14+ S100-	-CRTC1-MAML2 fusion -T (11; 19). (22)

## Discussion

Sclerosing microcystic adenocarcinoma (SMA) is a unique minor salivary gland neoplasm primarily affecting individuals with a mean age of 56 years and apparent female predominance (F/M: 9/5) (4, 5). A few reports of extracutaneous head and neck MAC in the tongue and parotid gland were published in 1995 (1, 6). Anne M *et al.* in 2016 reported five patients with MAC-like tumors arising in the mucosal surfaces of the head and neck (3). Analogous to this case with a history of high-grade lymphoma, most of the SMA cases had a background of malignancies such as adenoid cystic carcinoma, AML (7), ovarian mucinous cyst adenoma (8), nasopharyngeal carcinoma (9), squamous cell carcinoma of tongue (4), and two of them underwent head and neck regional radiotherapy (see also Table 1) (7, 9).

Microscopically, SMA is characterized by prominent stromal components intermixed with infiltrating nests and ducts of cytologically bland-looking cells. The stroma ranges from densely collagenized to basophilic-desmoplasia frequently surrounding the ducts. No specific molecular alterations have been identified for SMA, and the immunohistochemical profile is nonspecific. As a result, the diagnosis mainly relies on careful morphological evaluation and exclusion of other differential diagnoses. Histological diagnosis of SMA can be challenging, particularly in cases where small incisional biopsies are performed and the diagnostic features are not readily apparent (3). 

As an illustration, when the infiltrating pattern is absent in biopsies, the basophilic desmoplasia surrounding ducts might be confused with the sclerotic and atrophic lobules of salivary gland tissue; thus, it can be interpreted as benign. In addition, the pauci-cellular stroma of the tumor could be mistaken for reactive changes, especially when there is a history of a previous tumor biopsy (3). Histologic sections revealed mitotically inactive, relatively bland-looking small cuboidal to spindle-shaped cells arranged in discrete cords, isolated tubules, and bilayer strands of epithelioid cells forming microcystic lumina resembling reactive lesions, such as chronic sclerosing sialadenitis, the partially sclerotic stroma as well as desmoplasia, the highly infiltrative growth pattern of the tumoral cells invading normal salivary glands with extension to the deep muscular layers and perineural invasion, strongly argued in favor of malignant nature of the lesion (see also Table 2). 

Although the clinical impression was squamous cell carcinoma, this neoplasm lacked prominent cytologic atypia and surface epithelial dysplasia. In addition, some of the infiltrating tumor nests had a squamoid appearance (Figure 2f); nevertheless, the absence of keratin formation and the presence of ductal structures lined by cytologically bland-looking cells did not favor squamous cell carcinoma. 

The tubular pattern of the neoplasm and close association with minor salivary glands highly suggested salivary gland tumors, such as adenoid cystic carcinoma, low-grade mucoepidermoid carcinoma, and tubular pattern of polymorphous adenocarcinoma (PAC). The absence of angulated hyperchromatic nuclei and basophilic secretions, the presence of infiltrating small cords and nests of tumor cells, and negative CD117 (c-Kit) immunostaining ruled out the diagnosis of adenoid cystic carcinoma. PAC is usually more cellular and well-circumscribed at low power view with architectural variability; on the contrary, this neoplasm was widely infiltrative and paucicellular, containing abundant sclerosing stroma (see also Table 2).

Surprisingly deeper portions of this neoplasm were mainly composed of bland-looking signet ring-shaped cells arranged in cords, nests, and small ductal structures infiltrating interlacing striated muscle (intrinsic) bundles of the tongue parenchyma given the unusual and unexpected histopathologic presentation mimicking mucin-producing tumors like low-grade mucoepidermoid carcinoma, hyalinizing clear cell carcinoma, and Signet ring cell adenocarcinoma (see also Table 2). 

Signet ring cell (mucin-producing) adenocarcinoma of minor salivary glands represents 2% of minor salivary gland malignancies (10). In reality, this tumor may be more common than reported because it has not been universally recognized as a separate entity and has probably been diagnosed as adenocarcinoma "not otherwise specified" (NOS). Furthermore, Due to its morphology and mucin content, this tumor may be misdiagnosed as mucoepidermoid carcinoma, mucinous adenocarcinoma, or polymorphous low-grade adenocarcinoma (11) even though immune staining results revealed the glandular structures, which were positive for smooth muscle actin, P63, and P40 in the outer myoepithelial layers and CK7 in the inner epithelial components. The neoplasm's superficial and deep portions notably showed the same staining pattern despite their different cellular and morphological characteristics. Both myoepithelial and epithelial cells have undergone clear cell changes in the same way at the deeper portions of the tumor.

The final diagnosis was "sclerosing microcystic adenocarcinoma" with evident cell/signet ring cell changes after excluding the other more common salivary gland tumors. Other primary salivary gland tumors described with signet ring cell components are the more common ones, such as salivary duct carcinoma (12) and adenoid cystic carcinoma (13).

The association of SMA with immunodeficiency secondary to organ transplantation or hematopoietic malignancies has been reported, while MAC has been linked to solar radiation and therapeutic ionizing radiation for other causes. Interestingly, our case had a history of radiotherapy for poorly differentiated nasopharyngeal lymphoma, which may have contributed to the development of SMA and the clear cell changes observed in the tumor.

In summary, SMA is a rare neoplasm of the oral cavity with distinct histological characteristics. Its diagnosis relies on careful morphological evaluation and exclusion of other mimics. Our case underscores the challenges in diagnosing SMA, especially when encountering clear cell changes and atypical histological features. Further research and case studies for SMA are needed for a better understanding of the etiology, molecular alterations, and optimal treatment approaches.

## Conclusion

Here, we present a case of SMA of the tongue with clear cell changes in association with the past medical history of hematopoietic disorder and regional radiotherapy.

## Funding

None.

## Conflict of Interest

The authors declared that there is no conflict of interest.

## References

[1] Schipper JH, Holecek BU, Sievers KW (1995). A tumour derived from Ebner's glands: microcystic adnexal carcinoma of the tongue. J Lar Otol Lond.

[2] Petersson F, Skogvall I, Elmberger G (2009). Sclerosing sweat duct-like carcinoma of the tongue-a case report and a review of the literature. Am J Dermatopathol.

[3] Zhang R, Cagaanan A, Hafez G-R, Hu R (2019). Sclerosing microcystic adenocarcinoma: report of a rare case and review of literature. Head Neck Pathol..

[4] Wood A, Conn BI (2018). Sclerosing microcystic adenocarcinoma of the tongue: a report of 2 further cases and review of the literature. Oral Surg. Oral Med. Oral Radiol.

[5] Mills AM, Policarpio-Nicholas MLC, Agaimy A, Wick MR, Mills SE (2016). Sclerosing microcystic adenocarcinoma of the head and neck mucosa: a neoplasm closely resembling microcystic adnexal carcinoma. Head Neck Pathol..

[6] Zhang L, Huang X, Zhou T, Cao H (2020). Microcystic adnexal carcinoma: report of rare cases. Bioscience Reports.

[7] Jiang R, Marquez J, Tower JI, Jacobs D, Chen W, Mehra S (2020). Sequencing of sclerosing microcystic adenocarcinoma identifies mutational burden and somatic variants associated with tumorigenesis. Anticancer Res.

[8] Tan GZL, Goh GH, Loh KS, Petersson F (2021). Sclerosing microcystic adenocarcinoma of the parotid gland-The first recorded case with histo-cytopathologic correlation and a brief review of the literature. Ann Diagn Pathol..

[9] Lee Y-Y, Hwang T-Z, Jin Y-T, Chen C-C (2022). Sclerosing Microcystic Adenocarcinoma Arising from the Tongue: A Case Report and Literature Review. Diagnostics.

[10] van Weert S, van der Waal I, Witte BI, Leemans CR, Bloemena E (2015). Histopathological grading of adenoid cystic carcinoma of the head and neck: analysis of currently used grading systems and proposal for a simplified grading scheme. Oral Onco.

[11] Persson M, Andrén Y, Mark J, Horlings HM, Persson F, Stenman G (2009). Recurrent fusion of MYB and NFIB transcription factor genes in carcinomas of the breast and head and neck. Proceedings of the National Academy of Sciences.

[12] Xu B, Barbieri AL, Bishop JA, Chiosea SI, Dogan S, Di Palma S ( 2020). Histologic classification and molecular signature of polymorphous adenocarcinoma (PAC) and cribriform adenocarcinoma of salivary gland (CASG): an international interobserver study. Am. J Surg Pathol.

[13] Andreasen S, Melchior LC, Kiss K, Bishop JA, Høgdall E, Grauslund M (2018). The PRKD1 E710D hotspot mutation is highly specific in separating polymorphous adenocarcinoma of the palate from adenoid cystic carcinoma and pleomorphic adenoma on FNA. Cancer cytopathology.

[14] Ghannoum JE, Freedman PD (2004). Signet-ring cell (mucin-producing) adenocarcinomas of minor salivary glands. Am J Surg Pathol.

[15] Rooper LM, Argyris PP, Thompson LD, Gagan J, Westra WH, Jordan RC (2021). Salivary mucinous adenocarcinoma is a histologically diverse single entity with recurrent AKT1 E17K mutations: clinicopathologic and molecular characterization with proposal for a unified classification. Am J Surg Pathol.

[16] AlAli BM, Alyousef MJ, Kamel AS, Al Hamad MA, Al-Bar MH, Algowiez RM (2017). Primary paranasal sinus hyalinizing clear cell carcinoma: a case report. Diagn Pathol.

[17] Schwarz S, Stiegler C, Müller M, Ettl T, Brockhoff G, Zenk J (2011). Salivary gland mucoepidermoid carcinoma is a clinically, morphologically and genetically heterogeneous entity: a clinicopathological study of 40 cases with emphasis on grading, histological variants and presence of the t (11; 19) translocation. Histopathology.

[18] Saade RE, Bell D, Garcia J, Roberts D, Weber R (2016). Role of CRTC1/MAML2 translocation in the prognosis and clinical outcomes of mucoepidermoid carcinoma. JAMA.

[19] Hunt J, Stack Jr B, Futran N, Glass L, Endicott J (1995). Pathologic quiz case 1 Microcystic adnexal carcinoma (MAC). Arch Otolaryngol Head Neck Surg.

[20] Mills AM, Policarpio-Nicholas ML, Agaimy A, Wick MR, Mills SE (2016). Sclerosing Microcystic Adenocarcinoma of the Head and Neck Mucosa: A Neoplasm Closely Resembling Microcystic Adnexal Carcinoma. Head Neck Pathol.

[21] Bastaki J, Summersgill K (2010). Signet-ring cell (mucin-producing) adenocarcinoma of minor salivary glands: report of a case. Oral Surgery, Oral Medicine, Oral Pathology, Oral Radiology, and Endodontology.

[22] Kusafuka K, Maeda M, Honda M, Nakajima T (2012). Mucin-rich salivary duct carcinoma with signet-ring cell feature ex pleomorphic adenoma of the submandibular gland: a case report of an unusual histology with immunohistochemical analysis and review of the literature. Med Mol Morphol..

[23] Altemani A, Costa AF, Montalli VAM, Mosqueda‐Taylor A, Paes de Almeida O, León JE (2013). Signet‐ring cell change in adenoid cystic carcinoma: a clinicopathological and immunohistochemical study of four cases. Histopathology.

